# Factors influencing effectiveness in postgraduate medical education – a qualitative study of experiences of the responsible clinical consultants

**DOI:** 10.1186/s12909-018-1433-6

**Published:** 2019-01-03

**Authors:** Hanna Wijk, Sari Ponzer, Kristiina Heikkilä, Lars Kihlström, Jonas Nordquist

**Affiliations:** 1 Medical Case Centre, Department of Medicine (Huddinge), Karolinska Institutet, 141 86 Stockholm, Sweden; 2 Department of Clinical Science and Education, Södersjukhuset, Karolinska Institutet, Stockholm, Sweden; 3 Department of Health and Care Sciences, Linnaeus University, Kalmar, Sweden; 4 Department of Research and Education, Karolinska University Hospital, Stockholm, Sweden

**Keywords:** Qualitative research, Medical education, Leadership, Effectiveness, Professional development, Clinical consultant, Educational effectiveness, Postgraduate

## Abstract

**Background:**

Medical education leaders are important for educational quality in postgraduate medical education. Their work tasks are complex and contain different components. However, factors that are influencing leaders´ effectiveness in completing these tasks are unexplored. Understanding and developing these factors is most likely essential to strengthen postgraduate medical education and to consequently improve the quality in health care delivery. This study explores the experiences of factors that influence effectiveness of clinical consultants responsible for postgraduate medical education at clinical departments. Effectiveness was defined as fulfillment of work tasks.

**Methods:**

A qualitative study was performed with data gathered through semi-structured face-to-face interviews with 17 consultants responsible for postgraduate medical education. Data was analyzed by qualitative content analysis.

**Results:**

Findings clustered into four themes of factors influencing effectiveness: individual (being an expert, social competence), relational (support and cooperation, communication), attitudinal (shared vision, organizational values, colleagues’ attitudes) and structural (organizational characteristics, regulations and guidelines, conditions for the role). The factors were experienced to influence effectiveness in a positive or a negative direction.

**Conclusions:**

This study shed light on the complex and interrelated factors experienced to have impact on the role of consultant responsible for postgraduate medical education. Viewing the result through the concept of power, the role mainly relies on personal power sources like expert and referent power whereas power connected to the position often are lacking. To increase effectiveness of the role, a differentiated strategy which involves activities at both individual, group and organizational levels is needed.

## Background

Medical education leaders in postgraduate education are important for the quality of the education [1, 2]. Postgraduate medical education is organized in different ways around the world, but it often includes a formal position handling the postgraduate medical education program. Although the term for this position varies [3–6], in this study the term clinical consultant responsible for postgraduate medical education (CRE) is used. The tasks and practices of this position have been studied previously and have shown a complexity and variety of tasks including structuring the education, supporting individuals, handling relations, negotiating between different interests and influencing the educational culture [7]. The tasks have also been clustered into an intrapersonal, interpersonal, organization and systematic theme [4]. Malling, Scherbier and Ringsted [4] concluded that the expectations on the tasks and responsibilities of the role varied between stakeholders.

Leadership effectiveness has been a core topic in leadership research [8]. Previous studies have used a variety of criteria for effective leadership, with emphasis on group productivity and followers’ satisfaction [9]. Group productivity relates to the performance of the group lead by the leader, whereas followers’ satisfaction refers to subjective experiences of the persons led by the leader. Since there are several other criteria that have been neglected as a result of this emphasis on the mentioned aspects, criteria of leadership effectiveness have been identified as one of the areas for future investigations [8].

Traditionally, the primary focus in leadership research was on studying individual leaders [10], most often focusing on leaders’ behavior, resulting in a variety of different behavioral taxonomies [11]. Today the research area has broadened to include the link between leadership and for example organizational climate, followers and culture [12]. According to the cultural perspective on leadership, leadership is recognized as something that is co-produced by leaders and followers in a specific context. Culture is viewed as a result of this interaction, where behaviors are guided by contextual structures, routines, norms and rules [13]. In the domain of postgraduate medical education, the CREs have been studied from a leader-centered perspective, showing competencies for effective leadership [14], varying leadership styles [6] and the connection between leader behavior and educational climate [15]. However, contextual factors that may enhance or discourage the effectiveness of CREs are less studied, as is the relationship between the CRE and the context.

In summary, the importance of educational leadership in postgraduate medical education is established, as is, to some extent, the content of this role. Effectiveness of the educational leadership is an underexplored area, and there is a need for better understanding of the influencing factors. A deepened understanding would have the potential to influence organizational structures and regulations for the role of the CREs and the competence development of CREs regarding how these factors should be handled. Such an exploration could also serve as a basis for further studies within this area.

The aim of this study is to explore CREs’ experiences of factors that influence effectiveness of their work, both in a positive and negative direction. Effectiveness is here defined as the CREs’ fulfillment of work tasks. This definition was chosen since it enables an exploration of effectiveness including a variety of factors from the view of the CREs’.

## Methods

### Design

This is a qualitative study in which semi-structured interviews [16] were conducted in order to shed light on the CREs’ experiences. Data was analyzed according to inductive qualitative content analysis [17, 18] as instructed by Graneheim & Lundman [19]. Qualitative methodology was considered to be appropriate, since it is suitable for exploring a complex phenomenon, including the role of the context, and for understanding the phenomenon as it is perceived by people experiencing the phenomenon [20].

### Setting

This study was performed in Stockholm, Sweden. In Sweden, physicians’ postgraduate training can be delivered by all clinical departments and health care centers. According to regulations by the Swedish Board of Health and Welfare, CRE and supervisors are mandatory positions in the faculty. The CRE coordinates the internal and external education for the residents and provides support to the head of the department, the supervisor and the residents [21]. The role of CRE is managed as a part-time duty alongside work as a consultant, and can either include managing the postgraduate education within one department, or an overall coordinating role for several departments, for example an entire hospital. CRE most often reports to the head of the department, who is ultimately responsible for the education. Some departments have chosen to organize the role of the CRE to include a managerial position towards the residents, as well as the tasks and responsibilities of a CRE.

### Sample

A strategically purposeful sample using maximum variation sampling [22] was made among the CREs at two hospitals. The hospitals were chosen because they have CREs from different specialties, programs of varying size and different ways of organizing their position, leading to differences in experiences. Inclusion criterion was CREs managing postgraduate medical education at one department, thus CREs with an overall coordinating role were excluded. On an individual level, differences in gender, specialty and experience were sought, as well as respondents with and without a management position. Based on the above criteria, seventeen CREs were selected by the research team in consultation with administrators within the postgraduate medical education at the hospitals. The CREs were contacted by email, all agreeing to participate. The number of respondents was chosen since variation was obtained by these respondents and with a plan to contact further respondents if needed. After 15 interviews, no more themes in data were appearing.

### Data collection

Data consists of 17 interviews conducted at two different hospitals. Data was collected between February and August 2013. All 17 interviews were individual and were conducted by a psychologist (HW). A semi-structured interview guide [16] was developed, where the questions for this study related to factors that the respondents experienced to influence their ability to perform their work tasks. Pilot interviews with three CREs resulted in some minor changes in how the questions were posed. The pilot interviews were included in the material. The interviews lasted between 39 and 79 min, with an average of 55 min. The interviews explored participants’ perceptions about their tasks and which factors influenced how they could carry out these tasks. This study focused on the latter formulated with the overall questions “what are the obstacles that prevent you from carrying out these tasks in the best possible way” respectively “what enables you to carry out these tasks in the best possible way?” (Table 1, question 4 and 5). The two questions were asked several times for different tasks, which made the respondent talk about enablers and obstacles in parallel.

**Table 1:** Interview guide

1. On a general level, what is your assignment as a CRE?	
2. Describe the work tasks you carry out as CRE.	
3. What do you do in order to carry out these tasks?	
4. What are the obstacles that prevent you from carrying out these tasks in the best possible way?	
5. What enables you to carry out these tasks in the best possible way?	
6. Do you believe you have a leadership role as CRE?	
7. How do you perceive your role as a CRE?	

All interviews were digitally recorded and fully transcribed verbatim.

### Analysis

The data was inductively analyzed using qualitative content analysis as described by Granheim and Lundman [19]. The analysis process started with the first author reading through the interviews several times. Then, the text about factors influencing the effectiveness of the role of the CRE was brought out from the rest of the data. In the first analysis step, first and last author identified the meaning units individually for two interviews. Thereafter the identified meaning units were discussed and agreed upon and the rest of the interviews were then divided into meaning units individually by the two analysts. In the next step, the meaning units were coded, again individually, and also the codes were discussed between the two analysts. Thereafter, the various codes were compared based on their differences and similarities and sorted into categories in next step, and then, in the last step, formulated into themes. Table 2 gives examples of the alignment between different analytical units during the content analysis. To reduce the effect of investigator bias, the reasonability of the categorization and thematization was discussed within the group and consideration was given to any differences that may have occurred as a result of prior knowledge of the authors.

**Table 2:** Examples of the alignment between different analytical units

Meaning unit	Condensed meaning unit	Code	Category	Theme
And then he /head of department/ has also become interested – that is, he’s also interested in development and education. And he wants us to be the world’s best. So (postgraduate medical education) is an extremely important part of that.	Head of department interested in quality education at the workplace.	The head of department gives actively support.	Support and cooperation	Relational factors
I think that much of what hinders is that people see themselves or their own little section as an isolated activity, and refuse to see this overall and shared responsibility at the clinic regarding specialist training.	Colleagues see to their own section, not to the entire department’s interest regarding PGME.	Lack of cooperation within the department.	Support and cooperation	Relational factors
But much is that I have colleagues who are teaching minded, I think, whom I have managed to get on my side. It’s about getting them with me because they’ll have to manage this and carry it all on.	Colleagues are interested in education, follows me and carry on the work.	Colleagues wants to support and participate in the PGME.	Support and cooperation	Relational factors

In order to obtain credibility, we used the interview guide in two workshops with two different groups of CREs which were not part of the study population. The results from the workshops corresponded to the results from the interviews.

### Ethical considerations

Before the interview, the participants received written information about the purposes of the study, the right to withdraw from the study at any stage, and assurances about their anonymity. The information was also presented orally at the time of the interview. In addition, written consent was obtained from all participants. All interviews were performed in a private room with ensured privacy and confidentiality. Ethical approval for this study was applied for at the regional Ethical Review Board (dnr 2012/1662–31/5).

## Results

Seventeen CREs were interviewed. Table 3 provides a summary of the study population. The participants represented 15 specialties from a university hospital and from a teaching hospital. Each participant had worked as a CRE during various numbers of years, and the number of residents varied between 1 and 28.

**Table 3:** Summary of the study population

Characteristics of participating CREs *N* = 17	
Type of specialty	6 surgery
	6 medicine
	5 diagnostic
Type of organization	1 university hospital
	1 teaching hospital
Sex	8 men
	9 women
Years of experience	Range: 8 months – 12 years
Number of residents	Range: 1–28 residents
	Median: 12 residents
Type of role	12 CREs
	5 CREs with managerial position

From the data, ten categories were identified. Based on these, four themes (individual, relational, attitudinal and structural factors) were formulated (Table 4). The respondents were faster to identify factors that were hindering than enabling and spent more time talking about these factors.

**Table 4:** Factors influencing effectiveness

Categories	Theme
Being an expert	Individual factors
Social competence	
Support and cooperation	Relational factors
Communication	
Colleagues’ attitudes	Attitudinal factors
Organizational values	
Shared vision	
Organizational characteristics	Structural factors
Regulations and guidelines	
Conditions for the role	

### Individual factors affecting effectiveness

The first theme included factors linked to the CREs’ individual qualities. The CREs experienced that the individual factors primarily influenced their effectiveness in a positive way, as they gave them power to carry out their tasks.

### Being an expert

The CREs experienced their own expertise as a factor which affected the fulfillment of work tasks. They mentioned aspects like knowledge about educational topics (for example regulations and pedagogy) as well as clinical experience in the medical specialty. The latter was mentioned as connected to their own status at the workplace, which ultimately was experienced to affect their effectiveness. Other competencies that were experienced to affect the effectiveness of their role were competencies connected to the individual traits which made them function well as CRE.

“I am very structured. I am, I was the head of a clinic for a number of years, I’ve done all the training and evaluations, and I’ve gotten constant feedback on this, so it’s not just my perception of myself. And I think this is a good thing in contexts like this that involve long-term planning, or anything really, but in this case in terms of education programs.” (11)

### Social competence

The CREs described their ability to build a network and social relations as important for their effectiveness. Within their department, building a network with other consultants and the residents were mentioned. Externally, other CREs and heads of departments were specified as important. Some CREs described that they had intentionally built a social network, but for others this was explained as happening less consciously over time.

“But I believe I have a very good network of contacts, I know people everywhere. I’ve been working in Stockholm for twenty years. This is simply what I use, it’s my own personal resource.” (4)

The ability to have trusting relationships with the trainees was also viewed as affecting their effectiveness. This was described as an important factor, especially by the CREs without a managerial position.

This category is closely linked to relational factors, but differs in the way that the CREs described this as personal qualities and traits.

### Relational factors

CREs experienced relations with residents, heads of departments, supervisors and other consultants at the department as crucial for their effectiveness in the role as a CRE. Important aspects of the relations were support and cooperation as well as the quality of communication. The experience of the relational factors differed among different CREs; some found them to be positive for their effectiveness, others negative.

### Support and cooperation

CREs commented on the importance of having committed and cooperative supervisors and other consultants at the workplace. Some CREs experienced the supervisors as lacking commitment and interest in educational issues, which resulted in difficulties for the CRE in carrying out the work tasks. Others reported the supervisors as interested and as having a strong sense of responsibility for the postgraduate medical education. These CREs experienced their work tasks to be “easy” to carry out. Another person who was viewed as important in terms of support and cooperation was the head of department. Behaviors that were mentioned were practical help in difficulties, supporting the CREs’ work towards others in the organization and allowing the CREs to take their own decisions and actions.

“We (e.g. I and the department heads) can talk when something comes up. We can bring up issues, they listen and show understanding and such. (…) Yes, if something comes up, we bring up this and that, and discuss it, how we can do this or that.” (6)

In the cases where the CREs experienced a lack of support from the head of the department, they felt a lack of power connected to the role.

“I have no authority. I cannot say to the head of the department or anyone else either what to do. The head of the department has to tell the supervisors what to do.” (7)

### Communication

In addition to support and cooperation, the degree of communication with residents, supervisors and heads of departments was experienced as a key component for the effectiveness of CREs. The CREs mentioned effective communication to include regularity and continuity (e.g. meeting frequently enough), as well as a good representation (e.g. the right participants in meetings).

In addition to physical meetings, the CREs also experienced other communication channels as affecting their ability to perform their work tasks.

“I just find communication difficult. For example, for this supervisor course, oh! I sent out an email to this head of the department. I have identified on the list at the department that this and that supervisors haven’t gotten an advisor course (…) And then I got no answers. Then I sent it out again. And again, no answer. Then I spoke to the head of the department here, and she said, ‘yes, yes, I will answer. And still I didn’t get an answer. (…) So that I find difficult.” (17)

### Attitudinal factors

Attitudinal factors included CREs’ experience of others’ approaches towards postgraduate education and the CRE role. These were described as organizational values expressed by management and colleagues both in words and behaviors. Another aspect was the degree of shared vision for the postgraduate education.

Attitudinal factors were described by different CREs as either negatively or positively affecting their ability to perform the tasks of their role. These factors were described as closely linked to structural factors, in the sense that attitudes towards education were manifested in structural conditions within the organization.

### Organizational values

Organizational values regarding education and the role of the CRE were experienced as influencing priorities between education and clinical work, which were often stated as competing. For those CREs who felt that the organization showed that the role was highly valued, this was seen as an enabling factor for the performance of their tasks.

“The hospital’s board of directors is interested in shaping the CRE role…it feels like this is important, from the top.” (9)

### Colleagues’ attitudes

Closely linked to the organizational values were colleagues’ attitudes toward the role of the CRE. The experiences of this differed. Some of the CREs experienced that the role was seen as a responsible and important position, which resulted in positive relations and cooperation with colleagues in the faculty.

Others felt that the colleagues considered the role to have low status and low priority, which resulted in problems, for example with communication and cooperation. Also implementing decisions were difficult.

“I have to say that you are also meeting resistance of some part of the clinic (...) not everyone is respectful for this role.” (15)

Several of the CREs mentioned that because of the lack of formal power, the attitudes of the colleagues were crucial.

### Shared vision

The degree of shared vision for the postgraduate education at the department also influenced the effectiveness of CREs. This included shared views of the quality of the postgraduate education. In particular, the CREs mentioned that it was helpful if there were obvious developmental needs; that is, if coworkers were aware of poor educational quality that needed improvement, the vision was more likely to be shared. The CREs also experienced that another key to ensuring a shared vision was if the positive effect resulting from the work from the CRE was noticed in the workplace.

“My tool there was really this inspection (voluntary educational quality assessment) because there was nothing working at the department. Right there I had the tool to see just how terrible we are, we didn’t accomplish anything we were supposed to do, and then we could really emphasize the peer assessment, which is really an absolute criterion and demand.” (13)

### Structural factors

Structural factors included external structures that were experienced to set the frames for the role.

### Organizational characteristics

Organizational attributes*,* including financial resources, type of clinical activities at the department and organizational structure, were reported as important to the CREs’ ability to perform their tasks. Lack of financial resources was the most reported aspect when it came to counteracting the effectiveness of CREs.

“And then there are always the financial issues that come up, which is really difficult. That this doesn’t work, that’s not possible, they can’t get time to prepare and study, we can’t do this or that.” (10)

### Regulations and guidelines

Regulations and guidelines were most often experienced to have a positive impact on the effectiveness of CREs. These included, for example, regulatory decisions and workplace guidelines regarding postgraduate medical education. These were experienced to give the CRE more power to improve the postgraduate education and to carry out their tasks. An absence of regulations and guidelines was seen as hindering the effectiveness of the CREs, whereas complete and concrete regulations and guidelines were seen as enabling.

“So I think the papers are a support, I think the job description is a support, and that’s why they should be here. Anyway. So that we know what we’re supposed to do, or expected to do. It ensures, in some way, that this job is done correctly.” (13)

### Conditions for the role

Conditions for the role of the CRE included dedicated time for the assignment, competence development and mandate. Mandate contained, for example, aspects such as membership in decision boards and the right to decide about human resources such as scheduling and employment at the department.

The experience of the conditions of the role differed between CREs with or without a managerial position. The CREs with a managerial position to a greater extent found the conditions to be positive for their effectiveness, both in terms of time and mandate.

“The privilege is in a way, to be both manager and CRE. I've been able to drive educational questions in the management teams at a completely different way than if I had been only CRE.” (2)

CREs without a managerial position experienced the conditions for the role as having negative impact, both regarding lack of time and mandate.

## Discussion

This qualitative study provides a picture of the complexity of the inter-related factors that were experienced to influence CREs’ effectiveness in terms of fulfillment of work tasks. The results indicate that individual, relational, attitudinal and structural factors affect the effectiveness of CREs in performing their work tasks.

One way of understanding the material is to view it through the concept of power and its connection to effective leadership. Power is often defined as a person’s potential influence over events, attitudes and behaviors [8, 11], The use of the concept of power has been criticized both since it sometimes equates influence with power and because different bases of power are mixed together [8]. Several efforts have been made to understand power by making distinctions among different sources of power. French and Raven [23] have developed one of the most widespread taxonomies, including five different types of power. Table 5 shows the result mapped on these power sources.

**Table 5:** The result mapped on French and Raven’s bases of power

Type of power	Present in the study	Not present in the study
Legitimate power: comes from the position and job responsibilities a person holds	X	X
Coercive power: associated with people who are in a position to punish others		X
Reward power: based upon a person’s ability to bestow rewards		X
Expert power; comes from a person’s expertise	X	
Referent power: based on perceptions that the person is liked and respected	X	

Another distinction that is commonly used is the dichotomy between position power and personal power [24, 25]. The former includes potential influence coming from the person’s role in the organization, deriving from legitimate authority and control, over, for example, reward and punishment. The latter includes power based on, for example, expertise, friendship and charisma.

The findings revealed that the CREs experienced that effectiveness was closely linked to the experience of having power to influence the education. The CREs described having the possibility to influence through power sources deriving from their own competence as well as from their role in the social network. These factors, which were mainly experienced as positive for their effectiveness, represented personal power sources like expert and referent power.

Whether the CREs experienced legitimate or position power differed in our study. Legitimate power was experienced to be dependent on the attitudes towards the role of the CRE in the organization and among the colleagues as well as the type of CRE role: those with a managerial role described a higher level of legitimate power than those without this position. On the other hand, CREs without managerial role described the referent power, in terms of the trust and confidence from the residents and supervisors, as more important than did the managers.

A possible explanation for this emphasis on power can be that the CREs’ power over postgraduate medical education is problematic and that the authority for CREs often is lacking in the Swedish context.

A lack of position power like coercive and reward power has also been shown is congruent with the results from a study of educational leaders at the graduate level [26]. This may explain the necessity of support from, and communication with, the head of the department, who in turn influences the position’s power.

Another central aspect is the organizational and regulatory prerequisites, which are described under the theme of structural factors. The prerequisites are at all different levels, from the conditions for the individual CRE’s role, to the clinical health care structure at the department and hospital, up to regulations at an authority level. The factors that are experienced as most negatively affecting the effectiveness, including the lack of resources for postgraduate education and the conditions for the CRE, are among these prerequisites. The two aspects of power and prerequisites interact in a complex way, where a change in one theme also affected factors in the other theme; e.g. changed working conditions can increase power, and vice versa.

To maximize the effectiveness of leaders as well as other professionals, Karasek and Theorell point at the importance of a balance between the three parts: demand, control and support [27]. High demands and low control, on the other hand, predict low effectiveness and negative stress. Still, a high degree of social support can compensate for these risk factors. Our findings suggest the main factors hindering the effectiveness of the CREs are external and thus provides a low degree of control, and that the degree of social support shows large variations. Previous studies have shown a risk of burnout among educational leaders at postgraduate level [28, 29]. These results indicate that it is crucial both in terms of effectiveness and psychological well-being that the CREs’ sense of control is increased, and their social support strengthened.

### Limitations of the study

There are some study limitations. Firstly, the interviews were made in a specific context and may not be transferable to all other contexts. However, to increase the transferability a purposeful sampling of a diverse group of CREs was made, and the research context was thoroughly described. A second weakness is that the factors were exclusively explored from the view of the CREs. Further studies are needed to investigate the effectiveness of CREs from either an objective measure of educational quality, or a subjective measure based on the perceptions of others. Furthermore, the order of the questions may have affected the result, starting with hindering factors first time the questions were posed. However, the respondents own focus and often frustration over negative aspects affecting their role, suggests that the results probably would not have been affected if we have changed the order of the questions.A final potential weakness is the risk that researchers’ views on the topic from earlier experiences could influence the data analysis process. We reduced researcher bias by having several analysts with different experiences and by peer checking. Given these limitations, the strengths of our study include the focus on the CREs’ own experiences, the diversity of the sample and the links to the theories in the discussion, which we hope can be useful to discuss from and explore in other contexts.

## Conclusions

The effectiveness of the CRE is dependent on individual, relational, attitudinal and structural factors. The factors are complex and inter-related and may influence their effectiveness in a positive or negative direction. The findings illuminate that there are many critical changes in different domains that can be done to improve the effectiveness of the role.

Primarily, the findings underscore the need for a differentiated strategy that involves activities at both an individual, group and organizational level in order to ensure that the CRE has the power and prerequisites to carry out his or her work tasks.

The structural factors may be strengthened by mandate for the CRE, access to decision-making bodies and clear regulations.

The relational factors may be strengthened by routines with regular meetings with the head of the department, the residents and the consultants involved in training. Particular emphasis may be put on the development of a good relationship between the CRE and head of department. Commitment of supervisors can be influenced by structures and decisions that strengthen the symbolic value of education in the organization.

The attitudinal factors may be influenced by management sending signals about the value of medical education and CREs, for example in the different decision-making boards, financial priorities and in how time is allocated. Attitudes at the workplace may also be affected by starting from current problems in the education, formulating goals to redress these problems and following up on improvements.

The individual factors may be strengthened by continuous education to enhance the expert power, by professional network at the workplace, and by a thorough recruitment process when appointing new CREs. The findings may also have implications for the development of training programs for CREs.

Future research should investigate which factors are most essential for the quality of postgraduate medical education and should thus be prioritized in the work of strengthening the role.

## Abbreviations

CRE: Clinical consultant responsible for postgraduate medical education
PGME: Postgraduate medical education
